# Changes in functional brain networks and neurocognitive function in Chinese gynecological cancer patients after chemotherapy: a prospective longitudinal study

**DOI:** 10.1186/s12885-019-5576-6

**Published:** 2019-04-25

**Authors:** Yingchun Zeng, Andy S. K. Cheng, Ting Song, Xiujie Sheng, Huaidong Cheng, Yingwei Qiu, Jianfei Xie, Chetwyn C. H. Chan

**Affiliations:** 1 Research Institute of Gynecology and Obstetrics, The Third Affiliated Hospital of Guangzhou Medical University, Guangzhou, China; 2 Department of Rehabilitation Sciences, The Hong Kong Polytechnic University, Hung Hom, Hong Kong, China; 3 Department of Radiology, The Third Affiliated Hospital of Guangzhou Medical University, Guangzhou, China; 4 Department of Oncology, The Second Affiliated Hospital of Anhui Medical University, Hefel, China; 5 Department of Surgery, The Third Xiangya Hospital of Central South University, Changsha, China

**Keywords:** Gynecological cancer, Chemotherapy, Neurocognitive function, Functional brain networks, Chinese patients

## Abstract

**Background:**

Previous neurocognitive assessments in non-central nervous system cancers highlight the high incidence of neurocognitive dysfunction in this study population. However, there have been few studies exploring neurocognitive dysfunction induced by chemotherapy in gynecological cancer patients. This prospective longitudinal study was conducted to assess neurocognitive functioning and functional brain networks in Chinese gynecological cancer patients pre- and post-chemotherapy, while additionally including age-matched healthy subjects as the control group.

**Methods:**

All research participants were evaluated using a resting-state functional magnetic resonance imaging and neurocognition assessment. Behavioral data were conducted using SPSS for descriptive statistics, correlation and comparison analyses. Preprocessing of MRI (Magnetic Resonance Imaging) data and network analyses were performed using GRETNA (Graph Theoretical Network Analysis).

**Results:**

A total of 40 subjects joined this study, with 20 subjects in each group. With the exception of the mean of psychomotor speed, there was no significant difference pre-chemotherapy between patients and healthy controls in neurocognitive test mean scores (Ps > 0.05). During the post-chemotherapy assessment, there were significant differences in the mean scores of neurocognitive tests (including Digit Span tests, verbal memory, immediate recall, delayed recall, and information processing speed tests) (all Ps < 0 .05). Longitudinal graph analysis revealed statistically significant differences in the patient group, with significant decreases in both local efficiency (*P* < 0.01) and global efficiency (*P* = 0.04). Lower raw TMT-A scores were significantly associated with lower local efficiency (*r* = 0.37, *P* = 0.03). Lower verbal memory scores were statistically significant and associated with lower global efficiency (*r* = 0.54, *P* = 0.02) in the patient group, but not in the healthy control group.

**Conclusions:**

This study found that the risk of brain function and neurocognitive changes following chemotherapy could potentially guide patients in making appropriate treatment decisions, and this study may identify a cohort that could be suited for study of an intervention.

## Background

Gynecological cancer is the third most common malignancy among women in China [1]. While improving early cancer diagnosis and accessing effective cancer treatment increase cancer patients’ five-year relative survival rate, neurocognitive dysfunctions are significant sequelae of cancer [2]. Neurocognitive dysfunctions may affect executive function, psychomotor speed, attention and memory [3]. This can interfere with gynecological cancer patients’ capacity to accomplish activities of daily living, as well as with social and occupational functioning, leading to lower quality of life [2, 4, 5].

Advanced neuroimaging studies in cancer patients provide a better understanding of neurocognitive dysfunction after cancer treatment [6]. Previous neuroimaging studies have indicated changes in brain structure and function that correlate with neurocognitive function in gynecological cancer patients [7, 8]. While multiple neuroimaging studies have demonstrated structural and functional brain differences between cancer patients and healthy controls [9], structural changes in the brain cannot serve as a prompt or reliable biomarker for early diagnosis of treatment-induced neurocognitive disorders [10], as abnormalities in brain function usually appear before alterations in brain structure and clinical performance [11]. Certainly, some studies have demonstrated that structural changes co-occur with functional network differences [12], and the structure–function relationship is modality dependent [13]. Other research conducted on cancer patients also found that disruptions in brain structure and/or function may parallel [14]. Hence, detecting alterations in structural or functional brain networks might provide an earlier biomarker for neurocognitive dysfunction diagnosis [10].

Functional magnetic resonance imaging (fMRI) studies have shown that quantitative neuroimaging techniques, in combination with neurocognitive assessment, can be useful in advancing our understanding of treatment-induced neurocognitive dysfunction in cancer patients [7, 14, 15]. Different from task-dependent fMRI studies, resting state fMRI (Rs-fMRI) is task-independent and thus less vulnerable to confounds due to performance variance [16]. Rs-fMRI is a noninvasive neuroimaging technique that measures spontaneous brain activity [17]. Rs-fMRI does not require participants to engage in any cognitive activity, therefore providing unique advantages for clinical research studies [18, 19]. In addition, rs-fMRI is sensitive enough to measure intrinsic functional networks, which reflect various cognitive states, representing the majority of energy usage in the brain [20]. Thus, using rs-fMRI to detect brain changes pre- and post-chemotherapy is likely associated with aspects of disease and treatment pathology for cognitive dysfunction [14]. And utilizing a network analysis of rs-fMRI data, and linked neurocognitive changes with functional brain networks in cancer patients, would be promising to address the issue of interest in the present study.

The majority of neuroimaging studies on the neurocognitive functioning of patients treated with chemotherapy for non-central nervous system cancers have been conducted on breast cancer patients [10, 15]. Limited neuroimaging studies have been conducted on patients with gynecological cancer [7, 8, 21–23]. Given the poor understanding of the impacts of cancer and its treatment on neurocognitive function and functional brain networks in gynecological cancer patients, particularly Chinese cancer patients, it is important to explore any neurocognitive changes or functional brain network alterations in this population. Therefore, this prospective longitudinal study was conducted to assess the neurocognitive function, and functional brain networks, of Chinese gynecological cancer patients pre- and post-chemotherapy. The findings could add to the literature in meaningful ways by studying a cancer type that has received limited attention in terms of the cognitive and neuroimaging effects of treatment, while adding to the small body of literature that has examined these issues in non-Caucasian patient groups.

## Methods

### Subjects

The details of the study subjects have been previously described [24] but briefly described as Chinese adult women with a primary diagnosis of cervical, ovarian, or uterine cancer were ready for chemotherapy treatment. Age-matched women without cancer history were recruited as healthy controls. All age-matched healthy controls were recruited from among staff members at this hospital. This study obtained ethical approval from the ethics committees at both The Hong Kong Polytechnic University and The Third Affiliated Hospital of Guangzhou Medical University. All research participants joined this study voluntarily and provided written informed consent.

### Neurocognitive function assessment

The details of neurocognitive function assessment have been previously described [24] but briefly summarized as follow: this study took the recommendation of the International Cognition and Cancer Task Force (ICCTF): three core neurocognitive tests of the Hopkins Verbal Learning Test - revised (HVLT-R), the Trail Making Test (TMT), and the Controlled Oral Word Association Test (COWA) were used [25]. This study used the Chinese version of the Auditory Verbal Learning Test - revised version (AVLT-R) [26]; the Chinese version of TMT and the Chinese version of the COWA [27], respectively. As attention and working memory were the most common neurocognitive dysfunctions in Chinese gynecological cancer patients [28], this study also included the WAIS-III Digit Span test for measuring attention and working memory [29]. Cognitive performance testing in the patient group was conducted at post-surgery and post-chemotherapy, respectively. The duration of cognitive performance tests for patients were four months. As recruiting healthy controls needs to be age-matched with patients, it took two months to recruit eligible healthy controls, the duration of cognitive performance tests for healthy controls was two months. AVLT tests consisted of three successive learning trials and other procedures of cognitive tests were conducted consistently in both groups.

### MRI data acquisition

According to the ICCTF recommendations for neuroimaging studies in cancer and cognition, a minimal set of MRI sequences should include an rs-fMRI and a high-resolution T1-weighted anatomical MRI scan to assess functional brain networks [6]. Whole brain rs-fMRI data were collected on a Philips 3.0 T scanner (Achieva; Philips, Best, The Netherlands), using an 8-channel SENSE head coil at The Third Affiliated Hospital of Guangzhou Medical University, China. Throughout the rs-fMRI data acquisition, patients were instructed to close their eyes and relax, but to remain in a maximally alert state. A T2-weighted gradient-echo EPI sequence was used to obtain the rs-fMRI scan. A total of 240 whole brain EPI volumes were acquired using the following parameters: TR = 2000 ms, TE = 30 ms, flip angle = 90°, in-plane imaging resolution = 3 × 3 × 3 mm, in-plane field of view (FOV) = 256 × 256 mm, slice thickness = 4 mm, axial slices = 33. The rs-fMRI scan time was 8 min 6 s. T1-weighted imaging was achieved for morphometric (GM volume, cortical thickness and surface area) analysis using three-dimensional fast spoiled-gradient recalled acquisition in steady state (3D-FSPGR) in 164 coronal slices with the following parameters: acquisition matrix = 256 × 256; TE = 3.8 ms; TR = 8.2 ms; flip angle = 7°; FOV = 256 mm × 256 mm; slice thickness = 1 mm; voxel resolution =1 × 1 × 1 mm. The 3D-T1 scanning time was 5 min 58 s.

### MRI data preprocessing and network analyses

**Fig. 1:**
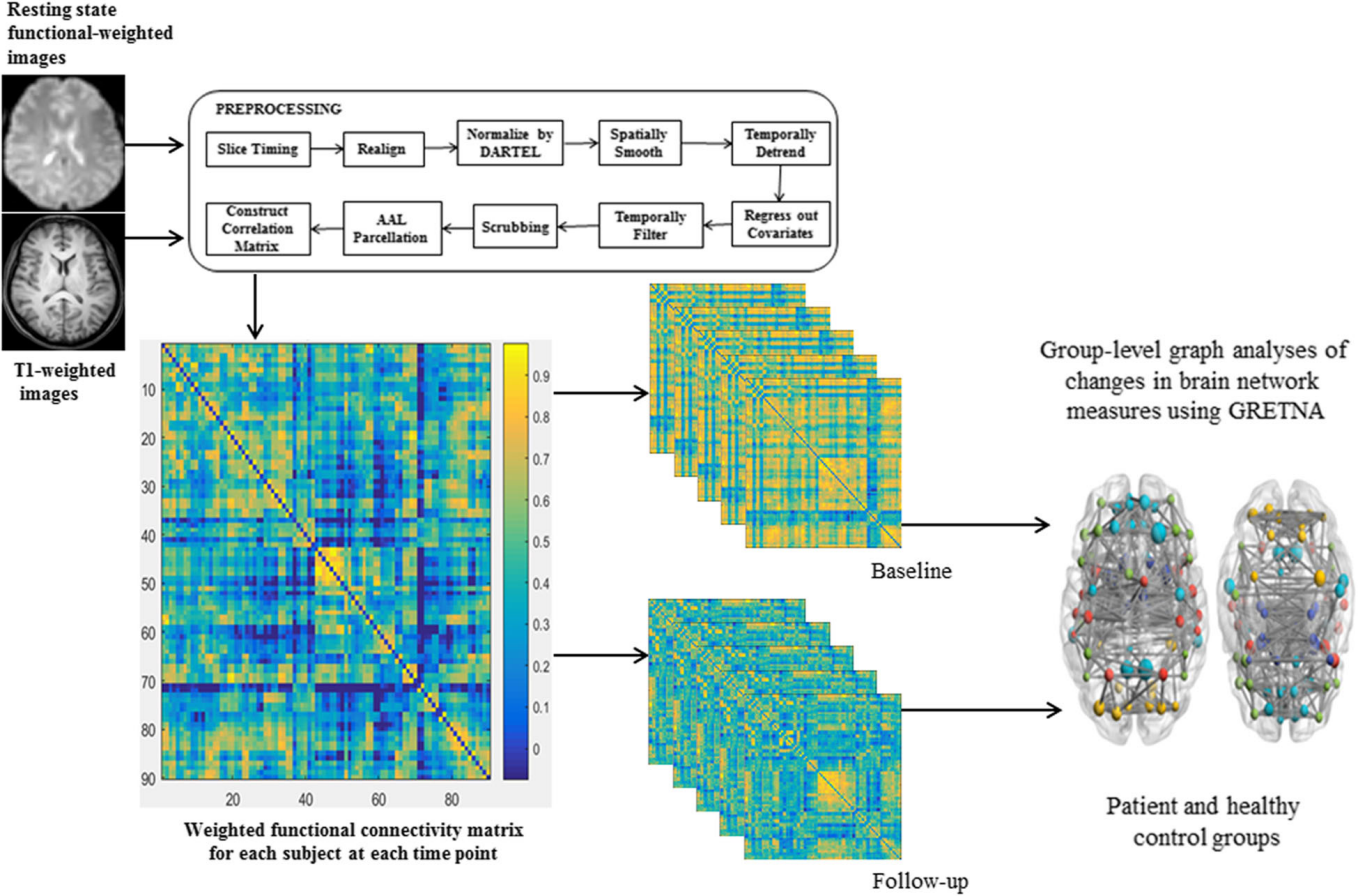
Illustration of brain functional network construction for longitudinal graph analysis

The rs-fMRI images were preprocessed using GRETNA: a graph theoretical network analysis toolbox for imaging connectomes [30]. During the preprocessing process, the first 10 volumes for signal were removed to reach a steady state, leaving 230 functional volumes for each subject. The remaining functional volumes were corrected for acquisition time delay between slices (slice timing) and head motion between volumes (realignment). Other steps in preprocessing these functional data consisted of spatial normalizing by DARTEL (warping individual functional images to the standard MNI space by applying the transformation matrix that can be derived from registering the final template file), spatially smoothing with a Gaussian kernel (full width at half-maximum of 4 mm), regressing out covariates (white matter, cerebral spinal fluid, global signals, and head-motion profiles are removed to avoid noise signals by multiple regression analysis), temporally linear detrending, temporal band-pass filtering (0.01–0.1 Hz), and scrubbing to reduce the effects of head motion on rs-fMRI data. The networks are constructed based on a voxel or region of interest approach. The Automated Anatomical Labeling (AAL) atlas was used to parcellate the brain into 90 regions (cerebellum excluded). Functional brain networks were constructed by thresholding the correlation matrices with a density of 5% [31]. All network analyses were performed using GRETNA [30]. The values were mapped onto the cortical surface using BrainNet Viewer [32]. Data preprocessing and network analyses are shown in Fig. 1.

### Hub identification

There are various methods to identify functional hubs. Some research suggests that hub regions can be defined as degree, betweenness centrality, and/or clustering coefficient values exceeding 1 SD (Standard Deviation) above the mean network, thus indicating hub status [33]. Other research indicates that nodes with a high degree, exceeding 1.5 SD above the mean network, can be identified as functional hubs, meaning that they exhibit high connectivity to the rest of the brain [31]. This study defined functional hubs of research participants with node degree values exceeding 1.5 SD above the mean network.

### Statistical analysis

Descriptive statistics, correlation and comparison analyses were conducted using SPSS for Windows (version 21; IBM SPSS Statistics, Armonk, NY, U.S.). Descriptive statistics are presented as mean, standard deviation (SD), and range. According to ICCTF recommendations, cancer patients were rated as experiencing cognitive dysfunction “if two or more neurocognitive tests (AVLT, TMT, COWA and Digit Span test) had a Z-score at or below -1.5, and/or one test had a Z-score at or below -2.0 of the healthy control group” [25]. Transformation of Z-scores was computed as subjects’ raw score minus the mean group score and divided by the standard deviation. Correlations of neurocognitive function with brain functional connectivity were made using Pearson correlation coefficients. Group differences were tested with independent or paired t-tests. All statistical tests performed were two-sided, and a *P* value of less than 0.05 was considered statistically significant.

### Validation analysis

To evaluate the robustness or reproducibility of the result, this study modified two key parameters of global signal regression and different connectivity density thresholds. For the validation of correlation between functional global metrics and associations with neurocognitive outcomes, this study used partial correlation analysis to adjust global signal as the covariate, as global signal of the whole brain is an important confounding factor for brain network analyses based on rs-fMRI [34]. Taken consideration of the connectivity density as an important parameter of the network topological structure, this study also validated study findings with network densities of 3 and 7% suggested by previous research [31]. A network null model for each group was designed with a density of 5% for comparison, as the weights of connections survived after thresholding with a density of 5% were applied.

## Results

### Research participant characteristics

Of 37 eligible patients, a total of 20 patients agreed to join this study and completed the baseline rs-fMRI and neurocognitive assessment. Four patients refused to attend the MRI scans and neurocognitive assessment post-chemotherapy. There were 20 healthy control subjects who were matched in terms of age, marital and menopausal status. The demographic and clinical characteristics of the research participants are summarized in Table 1.

**Table 1:** Demographic and clinical characteristics (*N* = 40)

Variables	n (%)	
	Cancer patients (*n* = 20)	Healthy controls (*n* = 20)
Age (years) Mean (SD)	47.15 (9.80) (28–60)	48.60 (6.80) (29–59)
Highest education		
Primary school or below	14 (70.0)	19 (95.0)
High school	4 (20.0)	1 (5.0)
University and above	2 (10.0)	
Employment status		
Employed	1 (5.0)	20 (100)
Unemployed	19 (95.0)	
Marital status		
Never married	2 (10.0)	1 (5.0)
Married	18 (90.0)	18 (90.0)
Divorced	0 (0.0)	1 (5.0)
Menopausal status		
Pre-menopausal	11 (55.0)	12 (60.0)
Peri-menopausal	8 (40.0)	7 (35.0)
Post-menopausal	1 (5.0)	1 (5.0)
Cancer type		
Cervical cancer	8 (40.0)	
Ovarian cancer	5 (25.0)	
Uterine cancer	7 (35.0)	
Disease stage		
Stage I-IIa	7 (35.0)	
Stage IIb-IIIa	8 (40.0)	
Stage IIIb	5 (25.0)	
Treatment type		
Surgery + Chemotherapy^a^	14 (70.0)	
Surgery + Chemotherapy^a^ + Radiation	6 (30.0)	

^a^Chemotherapy regimens including Paclitaxel (TAXOL) with Carboplatin (CBP) or Cisplatin (DDP) or with both; CBP with Doxorubicin (ADM) or TAXOL with ADM; Bleomycin with Methotrexate (MTX) or MTX with DDP

### Neurocognitive function of cancer patients compared to healthy controls

As illustrated in Table 2, with the exception of information processing speed, there was no significant difference at T1 (pre-chemotherapy) in the neurocognitive test mean scores between patients and healthy controls (Ps > 0.05). There was a significant difference in neurocognitive test scores, (including Digit Span tests, AVLT immediate recall and delayed recall, and TMT-A) (all Ps < 0.05) at T2 (post-chemotherapy). Transformation of patient Z-scores was computed as patients’ raw score minus the mean of the control group score at T1 and divided by SD. Z-scores of cognitive tests at T1 and T2 adjusted for education and employment status. From Table 3, there was a significant difference in Z scores of neurocognitive tests (including Digit Span tests, AVLT and TMT-A) (all Ps < 0.05) between patients and healthy controls at T1 and T2. There were seven patients at T1 and nine patients at T2 who reported cognitive dysfunction, respectively (Table 4).

**Table 2:** Comparison of cognitive testing mean scores between patients and healthy controls at T1 and T2

Variables	T1 Mean (Standard Deviation-SD)		*P*	T2 Mean (SD)		*P*
	Patients (*n* = 20)	Healthy Controls (*n* = 20)		Patients (*n* = 16)	Healthy Controls (*n* = 16)	
Attention and working memory						
Digit span forward	6.75 (2.53)	7.30 (1.92)	0.44	6.65 (2.55)	7.53 (2.03)	0.02
Digit span backward	2.45 (1.43)	3.15 (2.30)	0.25	2.40 (1.69)	4.26 (2.23)	< 0.01
Verbal memory						
AVLT immediate recall	5.32 (1.72)	4.63 (1.41)	0.43	5.10 (1.88)	9.15 (3.71)	< 0.01
AVLT delayed recall	4.95 (2.58)	4.45 (2.32)	0.52	5.01 (2.92)	7.35 (2.34)	0.01
AVLT recognition	10.35 (1.72)	10.40 (1.46)	0.92	9.55 (3.21)	10.40 (1.75)	0.31
Information processing speed						
TMT-A	57.65 (21.65)	44.95 (16.01)	0.04	54.20 (19.02)	38.83 (25.53)	0.04
Executive function						
TMT-B	71.05 (26.94)	57.80 (21.30)	0.09	73.35 (29.40)	56.72 (33.95)	0.11
Language						
COWA	33.65 (8.89)	31.55 (6.48)	0.31	15.55 (5.96)	25.65 (22.18)	0.36

*Abbreviation*: *AVLT* Auditory Verbal Learning Test, *COWA* Controlled Oral Word Association Test, *TMT* Trail Making Test

**Table 3:** Cognitive testing Z-scores between T1 and T2 among cancer patients

	Patients at T1 (*n* = 16)	Patients at T2 (*n* = 16)	*P*
Attention and working memory			
Digit span forward	−0.22 (1.16)	−0.32 (0.45)	0.04
Digit span backward	−0.25 (1.28)	− 0.08 (1.39)	0.02
Verbal memory			
AVLT immediate recall	0.81 (1.05)	−0.11 (1.10)	< 0.01
AVLT delayed recall	0.72 (0.86)	−0.48 (1.16)	< 0.01
AVLT recognition	0.42 (0.93)	−0.52 (1.11)	< 0.01
Information processing speed			
TMT-A	−0.14 (1.07)	−0.20 (1.08)	0.03
Executive function			
TMT-B	−0.19 (1.08)	−0.15 (1.02)	0.18
Language			
COWA	0.02 (0.88)	−0.04 (0.99)	0.13

*Abbreviation*: *AVLT* Auditory Verbal Learning Test, *COWA* Controlled Oral Word Association Test, *TMT* Trail Making Test

Z-scores adjusted for education and employment status

**Table 4:** Frequency of patients with decline on cognitive dysfunctions at T1 and T2

Cognitive domains	Patients with cognitive dysfunctions (No. %)	
	T1 (*n* = 20, 35.00%)	T2 (*n* = 16, 56.25%)
Attention and working memory	2 (10.00)	3 (18.75)
Verbal and learning memory	2 (10.00)	3 (18.75)
Information processing speed	1 (5.00)	1 (6.25)
Executive function	1 (5.00)	1 (6.25)
Verbal fluency	1 (5.00)	1 (6.25)

### Brain functional global metrics and associations with neurocognitive outcomes

All participants in the patient and healthy control groups demonstrated a small-world organization as indicated by small-worldness greater than 1. There were significant differences in small-worldness at T1 and T2 between patients and healthy controls (*P* = 0.04, and *P* = 0.02, respectively) (Table 5). There was a significant increase in characteristic path length at T2 between patients and healthy controls (*P* = 0.01). Results from the longitudinal graph analysis revealed a reducing trend of local and global efficiency in the patient group (Table 5). Lower raw TMT-A scores were significantly associated with lower local efficiency (*r* = 0.28, *P* = 0.04), and lower verbal memory scores were statistically significant and associated with lower global efficiency (*r* = 0.41, *P* = 0.03) in the patient group, but not in the healthy control group.

**Table 5:** Comparison of functional global metrics between patients and healthy controls at T1 and T2

	T1		*P*	T2		*P*
	Patients (*n* = 20)	Healthy Controls (*n* = 20)		Patients (*n* = 16)	Healthy Controls (*n* = 16)	
Small-worldness	1.63 (0.46)	1.89 (0.66)	0.04	1.55 (0.34)	1.84 (0.51)	0.02
Characteristic path length	0.98 (0.37)	1.12 (0.19)	0.28	1.32 (0.42)	0.96 (0.15)	0.01
Local efficiency	0.34 (0.03)	0.22 (0.09)	0.26	0.59 (0.38)	0.27 (0.05)	< 0.01
Global efficiency	0.21 (0.05)	0.24 (0.01)	0.64	0.17 (0.03)	0.25 (0.01)	0.45

### Characteristics of hub brain regions related to neurocognitive dysfunction

**Fig. 2:**
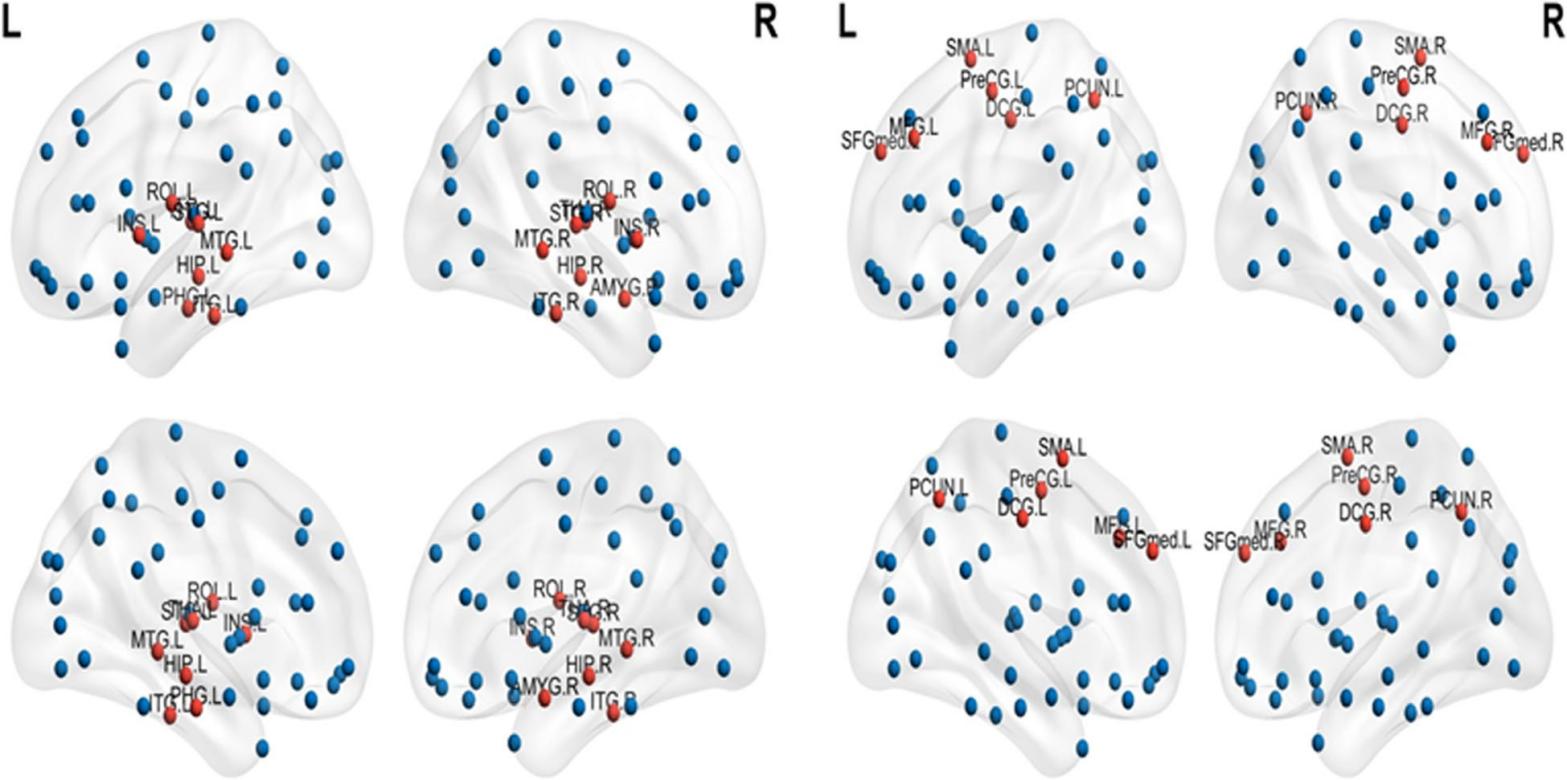
Hub brain regions (in red) of patients (left figure) versus healthy controls (right figure). L, left; R, right. AMYG, Amygdala; DCG,median cingulate and paracingulate gyri; HIP, Hippocampus; INS, insula; ITG, inferior temporal gyrus; MFG,middle frontal gyrus; MTG, middle temporal gyrus; PreCG, precental gyrus; PCUN, precuncus; PHG, parahippocampal; ROL, rolandic operculum; SFGmed, superior frontal medial gyrus; SMA, supplementary motor area; STG, superior temporal gyrus; THA, thalamus

**Fig. 3:**
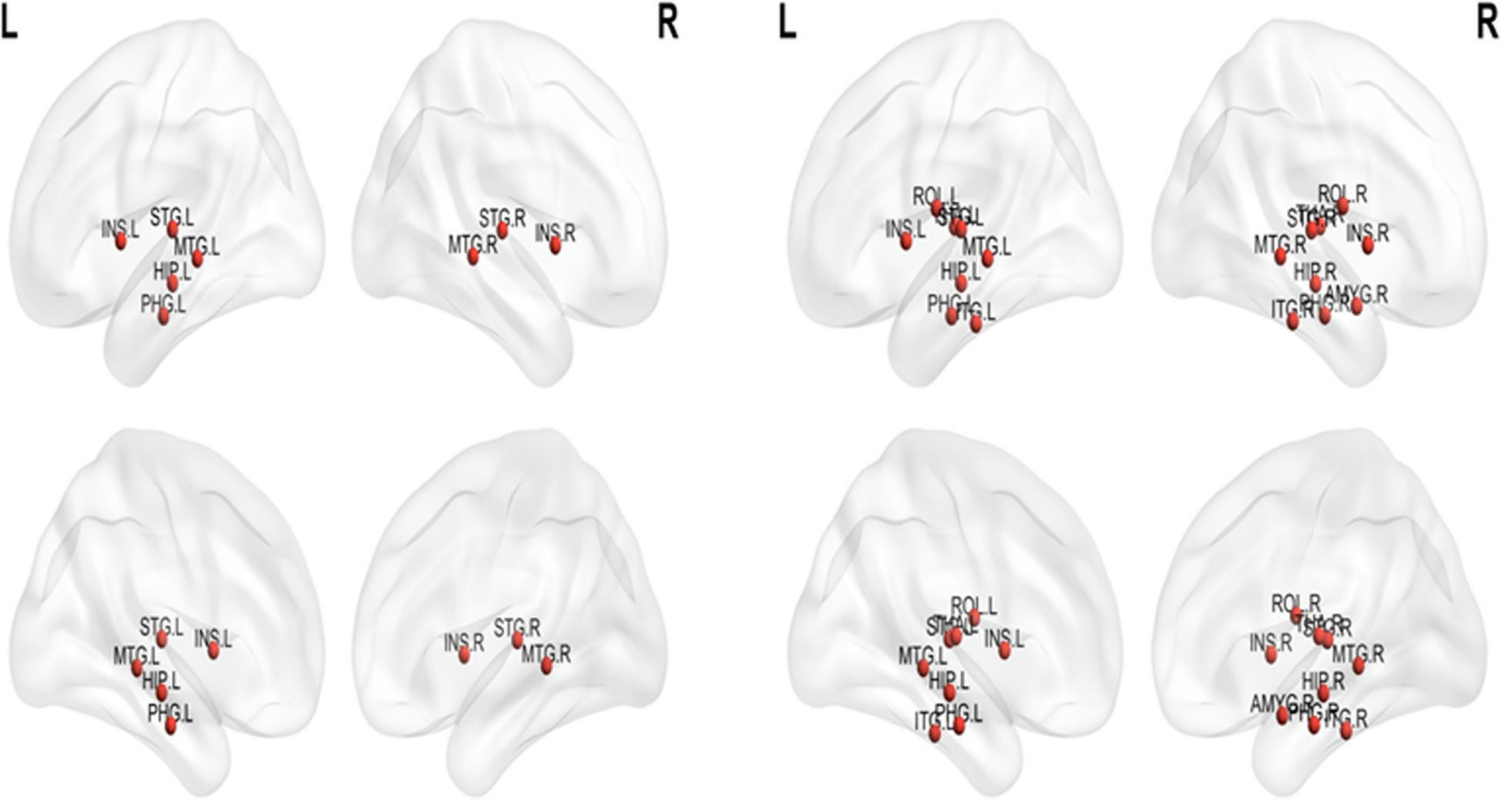
Hub brain regions of patients with cognitive dysfunction (left figure) versus patients without cognitive dysfunction (right figure). L, left; R, right. AMYG, Amygdala; HIP, Hippocampus; INS, insula; ITG, inferior temporal gyrus; MTG, middle temporal gyrus; PHG, parahippocampal; ROL, rolandic operculum; STG, superior temporal gyrus; THA, thalamus

Brain regions of research participants were evaluated for network hub status based on nodal degree values exceeding 1.5 SD above the mean network [31]. Hub characteristics of brain regions are shown in Figs. 2 and 3. As seen in Fig. 2, functional hub brain regions for cancer patients are mainly located in temporal regions, while parietal regions are the functional hubs in healthy controls. Within the patient group, left hippocampus, left parahippocampal gyrus, left and right insula; middle temporal gyrus, and superior temporal gyrus are functional hubs for patients with neurocognitive dysfunction (Fig. 3).

## Discussion

This is the first study to include a healthy control group with similar demographic characteristics, and a longitudinal design with repeated rs-fMRI assessment with the application of a longitudinal graph theoretical approach, to analyze brain functional networks in Chinese gynecological cancer patients. This study found that after chemotherapy treatment, gynecological cancer patients had lower neurocognitive test performance and changes in functional network measures, compared to age-matched healthy controls, which was in line with previous studies on cancer patients after chemotherapy [10, 14]. But the mean score changes of cognitive tests in the patient group were small in this study. It may be possibly due to surgery-related cognitive impairment, as all patients at baseline received treatment of surgery. Other research suggests that cancer patients treated with local surgery yield larger cognitive impairment than patients’ own baseline [35]. In specific, disrupted small-world properties were found in gynecological cancer patients. Functional networks with prominent small-world properties ensure higher information-processing efficiency for both locally specialized and globally integrated processing [36]. Decreased small-worldness index among cancer patients may result in lower information processing speed, which was supported by the significant associations of lower local network efficiency with lower raw TMT-A scores.

While the findings of this study indicated that the functional brain networks of both cancer patients and healthy controls show common small-world properties (both groups’ index values > 1), the local efficiencies were significantly higher in cancer patients post-chemotherapy than in the healthy controls. As local efficiency is a measure of average local subgraphs in a network, increasing local efficiency in cancer patients may result in disrupted information processing among distant brain regions [37], and lower network attack tolerance was associated with greater neurocognitive dysfunction in cancer patients [14]. In addition, this study found significantly decreased global efficiency, and significantly positive correlations between decreased global efficiency and lower memory scores, in the patient group only. Study findings were consistent with previous research, which reported reduced functional brain network efficiency in response to a simulated neurodegeneration in breast cancer survivors receiving chemotherapy, compared with healthy controls [15].

This study found that functional hubs were mostly located in the temporal regions for patients, and in the frontal and parietal regions for healthy controls, reflecting the main functions associated with these brain regions [36]. These study findings discriminated between the functional hub networks of patients and those of healthy controls, and also identified functional hubs for patients with cognitive dysfunction as well as for patients without cognitive dysfunction. Functional hubs for patients with cognitive dysfunction included the left and right insula, middle temporal gyrus, superior temporal gyrus, left hippocampus and parahippocampal gyrus, which are essential for network resilience and regulation of information flow [38], as functional hubs play key roles in forming bridges between different networks [39]. Brain regions with a high node degree were identified as hubs, which would be the most vulnerable areas in local functional networks [15]. Taken together, these findings suggest that all of these hub brain regions are key regions implicated in the pathophysiology of cognitive dysfunction; the connectome properties of these regions may to some extent predict neurocognitive functioning [15]. Therefore, this study’s findings provide new insights into the mechanism of cognitive dysfunction in cancer patients.

Evaluating the relative importance of brain neuroimaging features and their association with neurocognitive function was essential in understanding specific brain functional network patterns involved in neurocognitive dysfunction [15]. Rs-fMRI may be a particularly promising tool in identifying cancer patients at risk of long-term cancer-related brain injury [14, 15]. In addition, connectome metrics derived from rs-fMRI show good test-retest reliability [40]. Furthermore, the rs-fMRI acquisition required approximately eight minutes, making this scan a practical possibility in busy clinical settings. Thus, utilizing rs-fMRI could be a promising tool to better understand the longitudinal changes of treatment-related neurocognitive outcomes and functional network connectome properties.

The main limitation of this study is the small sample size, which may have reduced its power to detect functional differences between patients and healthy controls. This study found limited group differences achieving statistically significant differences in neurocognitive test performance, which may partially be due to limited power. Hence, in future research, there is a need to recruit larger sample sizes and use longer-term follow-up to replicate these results, and to investigate the potential reversibility of chemotherapy-induced changes [41]. In addition, the nongeneralizable convenience sample of this study may cause potential biases that could influence the conclusions. Finally, this study only chose the AAL atlas with 90 regions (ALL-90) as a brain parcellation scheme to calculate functional connectome properties, while excluding other brain parcellation schemes, such as Harvard-Oxford Atlas, as well as randomly parceling the brain into 1024 ROIs. According to previous studies on chemotherapy-related cognitive impairment in cancer patients [14, 15, 41], the AAL-90 parcellation is one of the most common brain parcellation schemes.

## Conclusions

Findings of this study have reported the first longitudinal evidence of brain functional network alteration and neurocognitive changes in Chinese gynecological cancer patients. This study found that information on the risk of brain function and neurocognitive changes following chemotherapy could potentially serve as a guide to patients in making appropriate treatment decisions, and this study may identify a cohort that could be suited for study of an intervention.

## Abbreviations

AVLT-R: Auditory Verbal Learning Test - revised version
COWA: Controlled Oral Word Association Test
CRCI: Cancer-Related Cognitive Impairment
GRETNA: Graph Theoretical Network Analysis
HVLT-R: Hopkins Verbal Learning Test - revised
MRI: Magnetic Resonance Imaging
TMT: Trail Making Test
